# Hospital Equipment

**Published:** 1914-07-11

**Authors:** 


### Hospital Equipment.

### A FIGHT FOR A KITCHEN.

No greater compliment could be paid to a manufacturing business than has been paid by the Dundee Town Council, through its Public Health Committee, to Messrs. James Slater & Co., Ltd., engineers, of London, W. New cooking appliances were wanted by the Dundee Town Council for the King's Cross Hospital, and, on the recommendation of the Public Health Committee, the contract of Messrs. Slater and Co. for £931 5s. 6d. was put forward for acceptance at the meeting of the Town Council on the 24th ultimo. This committee took the trouble to inspect ten other establishments and their kitchens, which led them to conclude that Messrs. Slater would serve the requirements of this department best. There was also the fact that Messrs. Slater and Co. gave a ten years' guarantee

Some of the Town Councillors put up a stiff fight against the acceptance of this contract, and a few Bailies, who favoured cheapness rather than quality, advocated acceptance of the lowest tender for £535. The Dundee Town Council, to their credit, stood firmly to the recommendation of the Public Health Committee, and in the end Messrs. Slater and Co.'s contract was unanimously accepted. This is a remarkable testimony, and we can support it from our own experience in regard to this firm's work, which we have examined many times and seen in hospitals and Poor-Law infirmaries up and down the country.

